# Assembling the salon: Learning from alternative forms of body work in dementia care

**DOI:** 10.1111/1467-9566.12461

**Published:** 2016-08-22

**Authors:** Richard Ward, Sarah Campbell, John Keady

**Affiliations:** ^1^ Department of Applied Social Science University of Stirling UK; ^2^ Department of Nursing, Midwifery and Social Work University of Manchester UK

**Keywords:** body work, care, dementia, hairdressing, embodiment

## Abstract

This article explores the labour and experiences of a hitherto entirely overlooked section of the dementia care workforce: care‐based hairdressers. Reporting on findings from the ESRC‐funded ‘Hair and Care’ project, the analysis and discussion focus upon the ‘doing of hair’ in the context of dementia care. The authors challenge existing assumptions and approaches to the management of appearance in dementia care, arguing for greater recognition of the subjective and culturally meaningful qualities of a visit to the salon. The article draws upon a wider debate on body work as a framework for the discussion, and considers the employment and working conditions of this largely hidden group of workers in the care system. The article offers an account of the praxis of care‐based hairdressing, with particular attention paid to narrative, intercorporeal and place‐making practices in the salon, showing how a particular approach to the body shapes the labour, relationships and activities that unfold within it. The authors argue that as an alternative form of body work much can be learned from hairdressing that can inform and enhance the provision of dementia care.

## Introduction

In this article we focus upon an entirely overlooked sector of the dementia care workforce – care‐based hairdressers. The focus here is upon the labour of the hairdresser and the conditions and wider context of the work. Our discussion draws upon the debate on body work and the opportunities this offers to frame our understanding of this largely hidden field of labour. As a particular form of body work, hairdressing provides a fruitful point of comparison to care practice, as both types of labour focus upon the bodies of people with dementia. Hence, close inspection and analysis of care‐based hairdressing can be approached as a means to interrogate the broader role of body work in dementia care. In a recent review of body work in the context of health and social care, Twigg and colleagues (2011: 171) define it simply as ‘work that focuses directly on the bodies of others: assessing, diagnosing, handling, treating, manipulating and monitoring bodies, that thus become the object of the worker's labour’. It is, they argue, both fundamental to social care and at the heart of the body pleasing, body pampering trades. A key feature of this type of work is the dynamic relationship between the way a body is understood and the nature of the work upon it: ‘Body work occupations appear to be shaped in the first place by definitions of the body which empower or constrain workers in relation to it’ (Wolkowitz 2006: 151). Consequently, bodies can be ‘reproduced’ in certain ways according to how they are worked upon, with questions of status and identity intimately caught up in this process.

A focus on body work has opened to scrutiny areas of (often feminised) labour that historically have been overlooked and consequently under‐theorised (Wolkowitz 2006). Hence Twigg *et al*. (2011) draw particular attention to the benefits of treating body work as a ‘linking concept’ in order to learn from examining the varying conditions and contexts in which it is undertaken. The contrasts and continuities between work that centres upon pampering and bodily adornment, compared to that involving the care of bodies, have proven a particularly fruitful ‘link’. Both are feminised fields of employment, where tensions between the unpredictable demands of bodies and efforts to rationalise the labour process create pressures of time at a practical level. Yet each involves a rather different worker/client dynamic; the ‘privileged’ body of the recipient of beauty work stands in marked contrast to the often stigmatised and disempowered body of the recipient of care.

### Dementia care and body work

Twigg (2000: 400) has suggested that the ‘official discourse’ which regulates and codifies care as a field of labour underplays the body to an extent that workers are ‘on their own’. Often seen as ‘dirty work’ Twigg argued that body work is hidden in such a way that the realities of working upon the bodies of others, those moments where we have to overcome disgust or discomfort as we negotiate such an intimate and taboo‐laden undertaking, are rarely written about. Lee‐Treweek (1997) similarly found that the actual doing of body work in care homes passed largely unobserved. She drew attention to the time‐pressured and sometimes coercive ‘behind the scenes’ bedroom encounters that prefigured the production of the ‘lounge standard’ resident. Lee‐Treweek revealed how an assembly‐line approach to care imposed a fixed standard of presentation on residents, functioning primarily to signify the quality of care provided. Thus, it was the suitably coiffed and neatly dressed ‘end product’ of this work that was subject to scrutiny within the care system rather than the doing of the work itself. Lee‐Treweek's analysis underlines how institutionalised efforts at the collective management of appearance erode the identities of individual residents, often imposing normative interpretations of femininity or masculinity in the process. In a recent review on embodiment and dementia, Kontos and Martin (2013) found that much of the commentary on body work in the context of dementia care underlines its role in the regulation and disciplining of the bodies of people with dementia. Far less attention has been paid to body work as a potential vehicle for empowerment or in the maintenance of the biographical self.

### Hairdressing as body work

The embodied dimension to hairdressing has similarly been neglected within research, often in favour of an emphasis on identities and social interactions within the salon (Yeadon‐Lee *et al*. 2011). Salons are spaces that are often age‐marked (Furman 1997, Twigg and Majima 2011) as well as upholding certain gendered, race and classed identities, and efforts have been made to analyse the hairstyle as a cultural artefact in respect to these intersections (Gimlin 1996, Mercer 1994, Tate 2009, Weitz 2001). However, through close scrutiny of styling and the embodied lived experience of hairdressers, Holmes (2015) departs from this earlier focus on the hairstyle as an endpoint to the hairdressing process by arguing for the importance of considering the ‘doing of hair’. Drawing on Sennett's (2008) notion of the ‘craft worker’, Holmes suggests that through the rhythm of routine and repetition, the practical skills of the hairdresser coalesce to become a craft. A distinction is made between skills, which are often defined by the level of formal education they require, and craft which is borne out of situated, embodied practice. Holmes (2015: 492) concludes: ‘the crux of the argument is that craft work occurs in occupations traditionally determined as lacking skill and expertise and producing “products” (objects, materials or bodies) of inconsequential value’.

### Hairdressing and the management of appearance in later life

While research has considered the role of hairdressing and beauty work specifically in later life, questions surrounding the meaning and management of appearance in late old age and in the context of illness or frailty remain largely unexplored. Gilleard and Higgs (2013: 130) commented recently on how difficult it is to find writing that deals with the efforts made to remain fashionable or ‘keep up appearances when corporeal limitations are present’. Instead, a preoccupation with illness and disability in social gerontology suggests the bodies of those in receipt of care are somehow positioned outside of culture (Katz 2011, Pickard 2013, Twigg 2010).

In the United States, efforts have been made to train hairdressers to notice the early signs of conditions such as dementia or depression and to impart advice on issues such as caring (Anderson *et al*. 2009, Solomon *et al*. 2004, Wiesenfeld and Weis 1979), thereby creating new frontiers for health and social care. Much less attention has been paid to the meanings that hairstyling and other aspects of beauty work hold for older customers or the tensions navigated between managing the ageing body and cultural expectations surrounding the need to ‘fight the signs of ageing’. Hurd Clark and Korotchenko (2010) and Ward and Holland (2011) have explored the politics of hair‐related practices in later life, highlighting experiences of social invisibility tied to greying hair and vulnerability to ageism as gender and ageing intersect at the level of the ageing body. Furman's (1997) exploration of a community‐based salon catering largely to older women in upstate New York similarly charts the complexities of ageing and ageism that inhabit beauty work, showing how the salon provides a space to redefine the experience of growing older, albeit against the backdrop of an ageist culture that intrudes upon how the older clientele view themselves.

The Hair and Care project is distinctive in examining a particular form of ‘body‐pleasing and pampering’ work as it is imported into the contrasting domain of body work as care, thereby positioning us to better understand the implications and outcomes for the people with dementia who are in receipt of both. Our argument here is that by recognising hairdressing as an alternative form of body work, the potential exists for learning and insights to emerge that are applicable to the organisation and enhancement of care.

## The study

The Hair and Care project was a 28‐month ethnographic study of appearance and the work of managing it in dementia care settings. Funded by the Economic and Social Research Council the study took place in north‐west England between 2010 and 2013. The overall aim was to generate a close and detailed description of the care‐based salon and the happenings within it, in the context of a wider consideration of appearance management in dementia care. To achieve this we pursued three more specific objectives:

- contextualising appearance through drawing on the perspectives of diverse stakeholders;
- exploring the embodied histories of people with dementia through ‘appearance biographies’ (see Ward *et al*. 2014 for further discussion of this method); and
- engaging with the immediacy of the body in the salon environment using ‘in‐situ’ and visual methods.

Ethical approval was obtained from an approved NHS Research Ethics Committee with knowledge of the Mental Capacity Act (2005) ID code: 11/WA/0147.

### Methods and analysis

Data collection consisted of a mix of qualitative methods that included mapping the provision of care‐based hairdressing across the area of the study (i.e. surveying the distribution and availability of hairdressing services and any gaps in provision). We conducted sensitising discussion groups followed by more in‐depth interviews with stakeholders (including care providers, people with dementia and carers). Diaries were kept by the researcher (SC) throughout the period of fieldwork. Filming and participant observation were used over a period of ten months in eight different care‐based hair salons in hospitals, day centres and care homes, as well as during visits to people's homes, and we followed a total of 23 people with a diagnosis of dementia (16 women and 7 men). During this time we amassed 48 hours‐worth of video footage and spent approximately 300 hours engaged in observation (spending between 3–8 hours a day in each setting).

We made a series of ‘process films’ to capture the patterns of activity and interaction in each salon, this involved filming as unobtrusively as possible in order to record day‐to‐day activity. We then progressed to filming a series of ‘in‐situ’ interviews, where we asked both worker and client to describe and discuss what they were doing as they were doing it (an approach that placed less emphasis on recall and memory). We carried out 13 ‘appearance biography’ interviews with people with dementia (7 women and 6 men), and semi‐structured in‐depth interviews with 10 hairdressers, nine family carers, six key informants (including a service commissioner, a hairdressing academic and dementia nurse specialist) and 15 care workers/nursing assistants. The interviews lasted between 20–130 minutes and on occasion took place over more than one session, for example as a response to someone showing signs of tiredness.

Different narratives served as the primary units of data for analysis (Riessman 2008). This included the practice‐related and experiential narratives elicited using in‐situ interviewing in the salon. We approached each narrative as a context that gave meaning to the events, practices and short stories enfolded within them (Phoenix *et al*. 2010). Narratives were also constructed using visual data generated for the project. For example, focusing upon the action surrounding sinks, hairdryers and mirrors we compiled accounts of how these material artefacts mediated salon‐based relationships. This layered approach to narrative supported our over‐arching aim of generating a rich description of the care‐based hair salon. Findings from the research were clustered into three interdependent themes. The first relates to the active role of the material environment in the hairdressing process, where the establishment of the salon required the translation of space into place. The second theme concerns the social environment that arose from the creation of the salon, and in particular the emergence of a distinctive salon culture. A third strand involved the embodied action and bodily collaborations that lay at the heart of the hairdressing process.

## Findings

### Situating hairdressing in dementia care

All the hairdressers we encountered in the course of our research were women, their pathway into the work shaped by a need to juggle caring commitments with paid labour. A number of hairdressers reported leaving salon work as their skills had become outdated, choosing to follow a certain age cohort of women throughout their career. All but one of the hairdressers was self‐employed with no formal contract, and consequently no sick leave or holiday cover. We found that hairdressers are rarely offered access to training by their host care‐providers, nor considered part of the care team and so do not participate in hand‐overs, have access to care files or receive briefings on newly‐arrived residents/patients. Yet, many had longevity of tenure, working in a particular care setting for anywhere up to 20 years, in direct contrast to the ‘churn’ of care staff in the same facilities.

The hairdressers spoke of a lack of clarity associated with their work, with host organisations largely silent over the detail of the hairdresser's remit. In practice, the line between hairdressing and care was blurred and this led to hairdressers taking *ad hoc* decisions over what they were prepared to do to meet the needs of their clients. Efforts to distinguish between different elements of body work hinted at the perceived boundaries to their labour: ‘I don't mind doing ears and beards, and eyebrows, I cannot do nasal [hair], just no’ (Hairdresser: HDR6). Tensions with the wider care regime were expressed through conflicting body work practices, for example, failure to align a client's weekly bath with a trip to the salon could mean they arrived with unclean hair or that their hairstyle would be washed out shortly after their visit. Once in the salon, hairdressers worked largely in isolation. As one hairdresser explained, this is intensive labour:

I had about ten [clients] yesterday, but it's hard work because lunchtime is in‐between and also I've got to do all the stuff myself. With big women or a big guy and they want to go to the bathroom and we're washing [hair] and the wheelchair's tipping over and I'm thinking there's nobody to help me sometimes, so that's difficult. It is hard work. Sometimes I'm thinking why am I doing this? Does anybody realise what I'm doing? … It does shatter you.(Hairdresser HDR2)

Limited disposable income for many clients tended to dictate pricing for the services on offer. In most salons, a shampoo and set was priced at between £8 and £10 (in 2013), which is roughly a third of the weekly Personal Expenses Allowance for care home residents in receipt of state aid. In all the salons, we witnessed an intense pressure on the hairdressers to balance the personalisation of each encounter with the need for rapid client throughput. In some of the larger care homes, hairdressers reported working with up to 30 clients in a day.

Working conditions were also a telling indicator of the status ascribed to hairdressing within the care system. It was surprisingly rare to find a designated salon in many care settings instead hairdressers were allotted a space on a temporary basis – perhaps the corner of a day room or a converted stockroom. As one hospital‐based hairdresser revealed healthcare environments were not designed with their service in mind:

I don't even have a room, I'm in the hallway doing their hair. I'm in the laundry. I'm in a room with a toilet when I'm cutting. And then there's no sockets in this laundry room, so I've got to move into the hallway with my hot tongs on the floor, so I keep my eye on them in case anybody comes.(Hairdresser: discussion group)

Rooms were often cramped which made manoeuvring wheelchairs particularly challenging and the available equipment was rarely designed for purpose. For example, we found many hairdressers using dining chairs to seat their clients and if access to a sink was available it rarely permitted wheelchair users to pull themselves up close for hair‐washing. Poor ventilation meant the dryers that were in constant use created a steamy almost tropical heat for the duration of a visit. Overall, hairdressing appeared neither integrated into the wider provision of care, nor well understood for what it offered clients.

### The labour of the salon

The hairstyling process itself followed a predictable pattern which shaped the rhythm of the salon: ‘welcome (sometimes with a short consult) – hair‐wash – rollers in (followed by setting lotion) – transfer to dryer – rollers out – combing out and final styling – ‘the reveal’ (sometimes using a handheld mirror) – goodbyes (occasionally preceded by payment)’. Standardisation of the labour was aided by the social homogeneity of the clientele. The large majority were women in late old age (i.e. 75 yrs+), all were white and according to the on‐going ‘salon chat’ it appeared that most if not all had followed a heterosexual lifecourse. Many also described growing up in working‐class neighbourhoods in the north‐west. Men were by no means entirely absent from the salon but were far fewer in number and their visit much abbreviated, usually confined to a quick trim with an electric shaver.

Yet, set against this backdrop of clients’ social proximity, the hairdresser was required to engage with corporeal diversity and immense variation in support needs. In this respect, the distinctive craft of the care‐based hairdresser lay in how she adapted her styling techniques and patterns of working to the myriad variations embodied by her clients, often doing so in the context of engaging three or four clients simultaneously. This was achieved through the careful integration of a range of different practices.

### Spatialising practices

The hairdresser's labour included spatial practices and incorporation of the material trappings of the salon environment into her work. On arrival the hairdresser was required to (re)create the salon, transforming a non‐descript space into a knowable place for her clients. Typically, most hairdressers carried much of their equipment around with them. Staging the salon involved re‐arranging furniture, often singling out a chair from a row and placing it strategically in front of a mirror or sink if either were available. Shelved trollies were positioned to each side and the accoutrements of the trade stacked into them; hairsprays and setting lotions, tubs of rollers, hairpins and hairnets, shaver and scissors. This staging process was vital in materialising the relationships that ensued, prefiguring the worker‐client dynamic. The singled‐out chair flanked by trollies intimated to arriving clients the one‐to‐one nature of the engagement that lay ahead.

Observation and filming highlighted how certain objects and material features played a distinctive part in the theatre of body work that extended well beyond their functional purpose and often emerged on the basis of how they were arranged in relation to one another. For instance, by placing a chair in front of a mirror and/or a sink, it would take on properties and qualities in the salon not previously possessed in the care setting, often becoming a micro‐site for self‐expression, self‐evaluation and the sharing of personal insights and memories. Hence, re‐arranging the care setting involved a process of re‐territorialisation, imbuing the space with fresh meaning as well as new functions.

Analysis of filmed footage drew attention to how the arrangement of furniture and equipment facilitated the flow of salon activity, providing material and spatial cues to clients. Staging the salon also reinforced a sense of place, creating clues that allowed clients to ‘place’ themselves – a process remarked upon by a number of care staff we interviewed. For instance, an occupational therapist on an assessment unit for people with progressed levels of dementia described her surprise in response to observing people in the salon:

Whereas in other activities of personal care or even meals and things like that, and medication, you'd have to give them lots and lots of cues and prompts – it's more of an automatic kind of reaction [in the salon]; they see what's there, they recognise what's there. Somewhere within that they have an awareness of what's expected of them, and they will sit down, they'll accept the towel round them, they'll adjust the towel […] and without being asked they'll bend their heads forward, and sometimes I'll go ‘oh!’ because it's so unexpected.(Occupational therapist hospital assessment unit)

This quote suggests that the creation of a knowable place in turn fosters the seamless and fluid performance of the part of salon client, but also highlights the significance of two bodies working closely together in familiar ways.

Availability of resources and equipment varied from one salon to another, however certain features were integral to practice. For instance, a wall‐mirror could be used to expand the hairdresser's presence serving as a tool for inclusion. She would use her reflection to engage with the client in front of her but also to monitor and interact with those awaiting their turn sat behind her, often pausing to catch a person's eye through the mirror before addressing them. Hairdressers used the chair as a ‘zone of personalisation’ and once seated many clients held forth: troubles‐telling; reminiscing and self‐analysing. The chair prompted storytelling, and could serve as a confessional, hovering at the boundaries of the public and the private. As a material prompt for certain types of interaction it supported the hairdresser's own efforts at coaxing stories from clients.

The hooded free‐standing dryer proved crucial in the hairdresser's struggle to rationalise her labour. The dryer could ‘hold’ a client while the hairdresser's attention was directed elsewhere. Once under the dryer its warming air and auditory hum created a cocooning effect, cutting the user off from the surrounding chat while relaxing her to a point of somnolence. A more ‘risky’ tool was the sink, which required clients to bend forward and allow water and suds to cascade over their face. As part of our experiential and embodied approach to fieldwork, Sarah participated in a hairdressing episode in a care home. Writing in her fieldnotes, she describes her own ambivalent response to this part of the process:

The hairdresser asked me if I was OK to face forward … She put a towel around me and I leant over the sink. There was lots of water rushing; it took a few moments for the temperature to become warm enough. The water rushed over me and I felt very wet, it was also very dark as I faced into the sink and couldn't open my eyes. I held the towel around my face. The shampoo massage was lovely, but I was very aware of all the water. The water was warm now, and the smells of the shampoo and conditioner filled my nose. As the hairdresser firmly lifted me up from the sink water dripped down my face. The light changed as I came up from the dark depths of the sink.(12.30 pm – 24 February 2012)

Slow‐motion video‐analysis also helped to reveal the intricate nature of sink work. Thermal, haptic and multi‐sensory experience is interlaced with apprehension and anxiety for many clients while the hairdresser combines touch and talk both to reassure and distract. Some clients were asked to count to ten, others strategically engaged in animated conversation and by taking advantage of her physical contact with clients the hairdresser could offer a reassuring back rub or accentuate the slow massaging of their scalp.

The spatialising practices of the hairdresser highlight an intertwining of the material environment with her embodied labour. More than simply tools of the trade the objects used extended her bodily presence and were synchronised to engage multiple clients, whilst maintaining a personalised service. By reconfiguring the lay‐out of the care‐space and importing equipment, products and instruments the hairdresser helps to instil a sense of place for participating clients. In part, this is achieved through material and multi‐sensory references to similar places from others times, including the many salons that a client may have visited over the course of her life.

### Fostering salon culture

Like the embodied practices of the hairdresser, ‘salon chat’ was both context‐dependent and context‐renewing – part of what made the salon, a salon. During interview hairdressers outlined their ‘special relationship’ with clients, functioning as confidante, enjoying a degree of intimacy which they claimed was rarely if ever extended to care staff. Vital to maintaining this relationship were the hairdresser's efforts at coaxing insights from her clients:

Hairdresser Do you feel better when you've had your hair done Pearl?

Client Yes, I do. My mother did bring me up properly I'm sure she did.

H I'm sure she did – was she a grand lady your mum?

C She was really yeh …

H Bet she worked hard didn't she?

C … and I was going to say she worked hard for all of us – she worked hard.

H What was your mother called?

C Vera.

H That's my grandma's name – Vera, and what was your father called Pearl?

C George.

H George and Vera … and what did your father do for a job?

C [pauses] Erm, he worked in the iron …

H In the ironmonger?

C No, making it.

H Like a blacksmith sort of thing?

C Yeh, like a blacksmith.

Hairdressers accumulated knowledge of a person's life over the course of repeated often weekly visits. Entrusted with personal insights, over time they became familiar with the well‐rehearsed stories clients told about themselves. Crucially, they functioned as the keeper of these stories; skilfully weaving in forgotten details or proffering opening lines to enable the continuity of conversation. In this context memory itself emerged as a shared and transactive process where stories were co‐constructed and the social presence of the client maintained as a result:

Client Leave my hair alone will yer, give over (waves her hand dismissively).

Hairdresser I can't stop halfway through, one side's flat and the other's frizzy.

C Oh never mind my hair, it makes no difference when you've done.

H (laughs) Yes it does! What did they used to call you when you were little?

C (pauses) … I … I don't know.

H What did you tell me people used to call you when you were little? (encouraging tone).

C (frowns) I don't know.

H Frizzy Lizzie! (smiles).

C Oh yes – they did! (smiles).

H They did didn't they – so I'm making all that frizz go away for you.

As this excerpt reveals, in supporting the recollections of their clients, hairdressers also use affective practices in order to gently manage the mood, often by adopting a buoyant tone and steering conversations into territory connected with happier recollections.

These supportive exchanges helped to foster and uphold a ‘micro‐culture’ within the salon; a means to carve out a distinct social space and experience within the wider care setting. Spoken and non‐verbal interactions acted as the carriers of salon‐specific values and etiquette providing a means by which to define the moral territory of the salon. For instance, the on‐going exchange of compliments was actively encouraged by the hairdresser: ‘What do you think ladies … doesn't she look lovely?’ This work of prompting and facilitating the acknowledgement of one another's appearance ensured each client was noticed and received attention during her visit, and was often coupled with efforts to counter their more negative self‐evaluations.

Central to salon culture was a collective investment in the idea of hairdressing as transformation. In this respect the hairdressers drew upon a fairly dominant trope within the beauty industry of body work as restorative and reinvigorating with powers that extended beyond surface appearance to affect wellbeing:

I did her hair and I cut it into a blonde bob, because it was naturally curly and I just blew it smooth and then cut it into a bob and the next thing she went like, put her skirt up, like you used to do at school, pulled her skirt up, turned it over [i.e. making a pleat to raise the hemline above the knee], got some red lipstick and she said come on we're going to the Swan [pub], me and you. I thought I daren't take her. But they all said ‘Well, I've never seen her smile for weeks’ and she felt so much better because her hair was done. And it lifts you. (Hairdresser: group discussion)

In the context of dementia care the transformative properties of hairdressing held particular significance for clients as an outcome of the hairdressers labour. Offering transition from one version of the self to another, clients were enabled to experience their bodies differently, consequently restoring the potential for more positive self‐narratives.

### Embodied practices

The supportive social interactions were mirrored by close bodily collaboration. For instance, throughout each visit the hairdresser signalled her intention to take responsibility for the multi‐sensory experience of her clients. She communicated this through on‐going enquiries into their comfort – ‘How does that feel?’; ‘Is it too hot or not too bad?’; ‘Bet that feels nice doesn't it?; ‘Tell me if this starts to pinch’ – and via close observation of non‐verbal and bodily responses. She would often anticipate less pleasurable episodes in order to closely manage the client's moment‐to‐moment bodily sensations.

A key outcome to the different embodied practices employed by the hairdresser was to enable individual clients to participate in the salon experience who would otherwise have been unable to do so. Hence, we witnessed how the hairdresser could use her own sensory acuity to supplement and substitute for the hearing and speech difficulties of her clients, engaging in the collective or shared management of impairment by using ‘inter‐sensory’ practices in order to facilitate social connections. The hairdresser would also combine her own strength with that of her client, finding the limits to their capacity and compensating for it. Often this was in response to a failure in the design of the salon to accommodate the diverse morphologies of her clientele. For instance, we observed and filmed Gloria in the course of a number of visits to the salon in the care home where she lived. What follows is an abbreviated account of the action surrounding her hair‐wash:

Sue the hairdresser assists Gloria to the sink which is set too close to the wall for her wheelchair to fit underneath. Sue wraps a layer of towels around Gloria's shoulders and front and then adds a plastic bib. She explains [to the researcher] this is necessary because Gloria has a ‘fuller figure’ and so finds it difficult to lean forward over the sink. She then adds another layer of towels over the bib. Standing behind the wheelchair she pushes the weight of her body up against it to leverage Gloria over the sink. Gloria responds by gripping the edge of the sink, pulling herself forward and tensing her body. Sue then reaches out and turns on the tap and grips the shower hose in her right hand while testing the water with her left. Once the water is warm she leans forward, her left elbow rests on Gloria's back and her forearm follows the line of her neck, culminating with the ball of her left hand pressing down on Gloria's head so that she faces into the sink. While pinning Gloria in this position Sue starts to massage her head with the fingers of her left hand while running the shower hose over her with the other hand. Stretching her neck out, Gloria offers muffled responses to Sue's checks on her comfort as the water bubbles through her hair. Maintaining her grip on the shower head Sue reaches out for the shampoo bottle and pours it into her left hand. She rubs the shampoo into Gloria's hair, massaging with her left hand and rotating the shower hose with the right. The water and suds flow down Gloria's face and onto the towel covering her chest as she strains to lean as far forward as she can. After rinsing, Sue steps back and allows the wheelchair to sit level. She quickly removes the wet towels and wraps a dry one around Gloria's head before starting to rub her hair dry. (HCP Salon 5: 20/3/12)

Such episodes of collaboration were an achievement of two bodies working together often involving close synchrony at key stages of the hairdressing process. The tempo of the labour created opportunities for worker‐client to syncopate their movements, responding to one another from moment‐to‐moment. Capturing these encounters on film underlined the shared forms of embodied agency that emerge during salon encounters.

### ‘Doing hair’ as intersectional practice

Close attention to styling practices and narratives in the course of in‐situ interviewing revealed that each hairstyle emerged from a matrix of intersecting forces that included bodily, material, environmental, cultural, biographical and economic considerations. For instance, hairdressers tended to avoid perming or colouring hair for clients with dementia due to their difficulties in tolerating prolonged waiting, while keeping hair short helped to prevent it getting caught up during lifting and handling in the receipt of care. Consideration of clients’ embodied experience beyond the salon visit was also a factor, as explained by one of the mobile hairdressers we shadowed as she visited a client with advanced dementia living at home:

With someone like Connie I go with the fact that she's got this illness and now she's in a chair. So she's leaning back all the time, she's sweating obviously in summer and things like that. She's sweating in her neck, so I keep her neck short for that reason. But I still like to go with a nice hairstyle, the way it flows really, and I cut it the way it wants to go. It's easier for the home‐help to just flick it into place and it will look nice, it will always look OK. Some of the home helps tend to comb the hair flat to the head, I just wish they'd just finger it and give it a bit of flow and let it dry, and it will dry curlier and wavier. (Mobile hairdresser HDR8)

The hairdressers understood that many of their clients were no longer able to tend to their hair but also that care workers gave less priority to hair‐care than to other aspects of body work. Concern for a client's image between visits was therefore a key influence upon styling and this was often coupled with an emphasis on offering value for money through ensuring the durability of a style:

Because I know like these days like blow waves are in, but some of them, like Jan, if you blow her hair, the day after it's just completely straight and she doesn't look nice. Whereas, if she has it set it'll look alright because she's not doing anything with it herself. (Hairdresser HDR6)

Here, the physical properties of hair itself are shown to play a role and many hairdressers talked of hair setting its own conditions on the cut (‘the way it wants to go’). During interview, one hairdresser demonstrated how hair can be lifted up and allowed to fall to reveal where the parting normally sits. Techniques like this could be used to ‘find’ a style for a client in a context where verbal communication was difficult.

While particular hairstyles often indicated broader age and class‐related preferences, these signifying qualities were combined with more immediate considerations associated with the material conditions of care. In this respect, care‐based hairdressers used body work to navigate the tensions between the more symbolic and expressive properties of hair and the constraints imposed by life in care. Indeed, their work underlines the role of appearance management in telling an on‐going story of the individual and their social situation. This narrative quality to appearance‐related practices was enabled through the joint workings and collaboration that are integral to salon‐based relationships and which taken collectively, helped to create a distinctive and culturally meaningful opening within the wider care system.

## The care‐based hairdresser's praxis: narrative – intercorporeality – place‐making

Our findings concerning care‐based hairdressing fit with the ‘bigger picture’ of body work as being largely hidden and often disregarded (Twigg 2000, Wolkowitz 2006). In the care system hairstyling is perceived as a low‐status service that delivers a transient and thereby ‘inconsequential’ product (Holmes 2015). Indeed, much as Twigg (2000) has argued of care, it is labour that is often only visible in its absence. We argue that such inattention to this distinctive form of body work has led to a failure to recognise its significance or achievements.

We found that storytelling was integral to the salon experience on a number of levels. Hairdressers actively participated in and supported the ‘storied selves’ of their clients, gathering snippets from regular conversations that effectively positioned them as a keeper of stories, able to facilitate their re‐telling often in the face of a client's progressive cognitive impairment. This process of ‘socially distributed remembering’ (Harris *et al*. 2014) has been explored with intimate couples living with dementia, for instance Hyden (2011) refers to the contribution of the spousal care partner as a form of interactional scaffolding that enables a person to perform well‐worn narratives. Yet, finding this level of support at the heart of paid labour is less common and points to the closeness of the worker‐client relationship, and the significance of the hairdressers’ role in the lives of their clients.

We discovered that the narrative practices of the hairdresser extend beyond the realm of ‘salon chat’. Storytelling can be an embodied‐enacted process, and this takes on heightened significance where people start to lose command of spoken language (Hyden 2013). In this context, the on‐going work of appearance management can function as an important outlet for self‐expression. Commentators such as Miller (2010) and Twigg (2010) have, for instance, suggested that dress is integral to an on‐going narrative of the self, where appearance gives outward form to the biographical self (Giddens 1991). Building on this metaphor, we argue that appearance‐related practices such as hairstyling constitute the ‘doing’ of this materialising narrative (see Ward *et al*. 2014). Crossley's (2006: 104) notion of ‘reflexive body techniques’ is useful here as he refers specifically to those practices which ‘work back on the body so as to modify, maintain or thematise it in some way’. He argues that such practices are vital in moulding our sense of self, and a relationship with the worlds we inhabit. According to Crossley, hairdressers belong to a small circle of body workers who participate in this reflexive relationship, and as such we would argue play an important role in the co‐construction of our embodied narratives.

Care‐based hairdressers are required to balance the more symbolic properties of hair with awareness of the material conditions of care and the bodily limitations of their clients. Hence, their work is not only concerned with preserving or maintaining aspects of self and identity (expressing the gendered, classed and age‐related preferences of their clients), but is a situated achievement, helping to tell a story of that person in the here and now. Understanding body work in this way as a form of ‘embodied narrative support’ casts it in a rather different light to the instrumental and task‐oriented approach to appearance work that currently characterises much care provision (Cohen Mansfield *et al*. 2006, Ward *et al*. 2008). Indeed, in a field where narrative practice has long been argued to have therapeutic potential (e.g. Baldwin 2008, Keady *et al*. 2007, Roach *et al*. 2014, Robertson 2014), our analysis points to how body work may be integrated into this endeavour.

Differing levels of capacity were collectively and relationally managed or renegotiated in the salon. Hairdressers used their own sensory acuity to facilitate interaction and social connections that would otherwise not have succeeded; they worked closely to co‐produce a pleasurable multi‐sensory experience of the salon; and used their bodily strength to augment that of their clients. Commenting on the nature of the stylist‐client relationship Cohen (2010a) argues that employment conditions can be an important influence. Hence, freelance and mobile workers who depend upon the loyalty of their clients seek to foster a bond to ensure repeat business, under conditions where it ‘pays to be friendly’. Close working with a client to ensure their collaboration and cooperation also reduces what Cohen (2011) describes as ‘baggy’ (i.e. wasted) time where body time and clock time diverge. Yet, we argue that this close sensory and bodily intertwining in the salon signals more than the instrumental efforts of workers to rationalise their labour.

Commentators such as Crossley (1995) and Weiss (1999) argue for the importance of recognising intercorporeality as fundamental to our social experience. Intercorporeality refers to the interdependence of bodies as they work together to negotiate the contingency and contextual variability of everyday life. Weiss argues that attention to the intercorporeal has the potential to challenge our most basic conceptions of the body and body image. Indeed, the range of embodied, inter‐sensory and affective practices that we witnessed, alongside the shared remembering and transactive nature of memory on display in the salon, proved vital in maintaining clients’ social presence and their continued participation in a salon experience that has punctuated much of their lives.

Building on these perspectives Price and Shildrick (2002) foreground the permeability of bodies in a context of their own ‘differently embodied’ relationship. Their argument is that bodies are always in process and open to negotiation through our relations with others. Their critique concerns the ‘disabled/able‐bodied’ binary that underpins a bio‐medical discourse where ‘bodily difference – read as impairment – is positioned as a problem [for one individual]’ (Price and Shildrick 2002: 67), rather than being shared in the negotiation between bodies. Zeiler (2014) further develops this argument specifically in relation to supportive relationships involving people with dementia, arguing that capabilities can ‘spring forth’ during intercorporeal encounters. Analysing a filmed episode using song and rhythm to engage a person with severe dementia, Zeiler highlights the ‘asymmetrical vulnerability’ that exists in such encounters and yet, through coming together to create ‘intercorporeal capabilities’, the individuals concerned form a ‘joint interface’ with the world. Our findings suggest that such joint capabilities lie at the heart of care‐based hairdressing, albeit in a more mundane fashion, and their accomplishment signals how hairdressers understand the body in the context of their work and enact that understanding, as an embodied way of knowing.

Paying attention to intercorporeality also has implications for our broader understanding of body work which is often characterised as labour done ‘to’, ‘on’ or ‘for’ a body by another (Twigg *et al*. 2011). Such an approach, we argue, downplays the contribution and agency of the recipient of body work and the interdependence which lies at the heart of much of this work. Observations in the salon highlighted the at times concerted efforts of clients to ensure the success of the hairdressing process. For instance, witnessing Gloria tensing her body while maintaining a firm grip on the sink and straining to hold herself in place throughout her hair‐wash, demonstrated clearly that body work is an intercorporeal achievement, determined by the contributions of both parties. Indeed, as a focus of their combined efforts, the hairstyle itself emerges as a unique ‘intercorporeal artefact’ which is testament to the intertwining of bodies in the care‐based salon, playing a vital role in an on‐going embodied narrative of identity and belonging.

In addition we learned that body work is a spatialising practice. The hairdresser assembles the material conditions that provide the basis for certain forms of conduct and interaction to unfold and, for many participants this supported the fluid performance of their role as salon client. In part, this is achieved through the strategic placement of objects and items that serve to materialise the relationships the worker intends to pursue. For instance, the chair positioned in relation to the mirror invites pause for thought and the sharing of biographical narratives, in a way that underlines the relational quality of objects and how they interact with one another, to create the ‘theatre’ of body work. As Rowsell (2011: 335) has argued ‘the situating of objects and artifacts‐in‐place can be as important as analyzing their materiality’. Crucially, such spatialising practices provided continuity for distinctive patterns of social interaction, conduct and activity.

Manipulation of space also had implications for the temporalities of the salon. In her work on high street salons, Black (2002: 5) argues that the offer of ‘time out’ and ‘time for the self’ lies at the heart of the salon experience: ‘Time here is the commodity being bought in the salon, and pampering is the means by which it is filled’. Yet, as Cohen's (2010a, 2010b) analysis of mobile hairdressing shows, when a hairdresser steps out of the salon, for example, to visit a client at home, she can lose temporal control with implications for the economic viability of her labour. In the case of care‐based hairdressers, efforts to reinstate temporal control were very much tied to their efforts in (re‐)assembling the salon. In this respect our focus on the ‘doing of hair’ as emplaced labour proved valuable in understanding the relationship between body work and a ‘wider ecology of things’ (Pink 2011: 345). It helped us to recognise how bodies are parts of places, but also in following Pink's analysis, led us to rethink ‘place’ itself as a spatio‐temporal phenomenon or ‘event’.

This notion of a place‐event (which draws on the work of Massey 2005) is apposite to our analysis of the salon as both spatially and temporally distinct from the care settings out of which it is carved. In order to achieve this, the hairdresser not only imports into care the accoutrements of the salon with their attendant and evocative sensory properties but also fosters an alternative temporality; one of ‘time out’, ‘time for the self’ and crucially ‘time with’ that lies at the heart of the beauty trade. Through their spatio‐temporal practices care‐based hairdressers thereby reveal the malleability of the care‐space, and the agentive possibilities that reside within the ‘place‐event’ when people with dementia are supported in collective forms of place‐making to redefine the environments that they inhabit. In this respect, the salon exists as an accumulation of embodied, relational practices and is actively constituted through the participation of people with dementia.

## Conclusions

The picture of care‐based hairdressing (CBH) that emerges from our research has implications for policy, practice and for research. For policy, CBH remains hidden labour, poorly understood despite clearly making an important contribution to ‘living well with dementia’ (DoH 2009). Combining elements of both care and hairdressing it is a distinctive field of labour but unrecognised as such in terms of training and support by care providers or the hair and beauty industry. This means there is currently no debate about or understanding of what represents good practice or the range of potential outcomes for clients who use this service. Wolkowitz (2006) points out that body work is often ‘bracketed off’ in discussion of the labour market, and the need to raise the profile for this sector of the care workforce was a key message from the hairdressers we consulted with during the life of the project. Only then can we more fully recognise the significant outcomes of this work as it supports continuity of identity through social interaction and acts as a buffer against certain unwanted effects of bodily ageing and ill‐health.

In relation to practice, we found that hairdressing is rarely embedded in the wider therapeutic activities of dementia care and individual workers reported at times unwelcoming and marginalising responses to their presence in the care system. Yet, by recognising that both hairdressing and care are fundamentally forms of body work, the potential exists to exchange knowledge and good practice. CBH can help us to radically re‐think approaches to the collective management of appearance in care, offering a framework for understanding and responding to the culturally and subjectively meaningful nature of grooming‐related practices. Indeed, it holds out the potential to interrogate and problematise existing conceptions of the bodies of people with dementia that currently drive dementia care, pointing to an alternative rationale for the body work of care as a vehicle for empowerment and maintenance of the biographical self.

Finally, our study illuminates insights relevant to a wider debate on body work. Using visual methods we captured the intricacies of the labour, underlining the skills or ‘craft’ involved, and illustrating the complex ‘ensemble of practices’ accomplished as worker and client pull together. Such insights highlight the importance of close scrutiny of the actual doing of body work as a route to understanding how different ways of working on the body have direct implications for the status and identity both of workers and the recipients of their work. We found that importing an alternative way of understanding the body into the domain of an established field of body work, has an almost subversive and hence transformative quality. Hairdressing ‘ruptures’ the care setting by introducing or re‐connecting clients to differing ways of understanding and using their bodies, that in turn open doors to alternative self‐narratives. This teaches us the importance of attending to the context and conditions of body work, appreciating its meaning as a situated accomplishment, and ultimately of recognising it as a significant form of place‐making in the lives of those it supports.
